# Online guided meditation training (Isha Kriya) improves self-reported symptoms of anxiety and depression within 2 weeks of practice—An observational study

**DOI:** 10.3389/fpsyt.2022.944973

**Published:** 2022-09-23

**Authors:** Sepideh Hariri, Ramana V. Vishnubhotla, Preeti Upadhyay Reed, Akila Rayapuraju, Hibiki Orui, Pavitra Balachundhar, Senthilkumar Sadhasivam, Balachundhar Subramaniam

**Affiliations:** ^1^Sadhguru Center for a Conscious Planet, Department of Anesthesia, Critical Care and Pain Medicine, Beth Israel Deaconess Medical Center, Boston, MA, United States; ^2^Department of Anesthesia, Harvard Medical School, Boston, MA, United States; ^3^Department of Radiology and Imaging Sciences, Indiana University School of Medicine, Indianapolis, IN, United States; ^4^Department of Anesthesiology and Perioperative Medicine, University of Pittsburgh School of Medicine, Pittsburgh, PA, United States

**Keywords:** meditation, anxiety, depression, Isha Kriya, online

## Abstract

**Introduction:**

Anxiety and depression have increased dramatically 2–3-fold with the COVID-19 pandemic. There is an urgent need for safe, cost-effective, and scalable approaches to alleviate this parallel mental health pandemic. Meditation has previously been shown to reduce stress, anxiety, and depression symptoms. Furthermore, online delivery of mind-body interventions will be impactful in addressing disparities in access to mental healthcare. In this observational pilot study, we investigate the impact of a digitally delivered guided meditation followed by daily practice on symptoms of anxiety and depression.

**Methods:**

Initially, 57 male and 202 female subjects enrolled in this study. Participants attended a webinar where they learned the Isha Kriya meditation practice. They were subsequently requested to perform the intervention daily for 6 weeks. Subjects were given scales to assess anxiety and depression at baseline, 2, 4, and 6 weeks following the training. The changes in the self-reported anxiety and depression scores were examined by the linear mixed effect models.

**Results:**

Participants completed survey responses for the following time points: baseline (*n* = 82), week 2 (*n* = 58), week 4 (*n* = 37), and week 6 (*n* = 28). During the 6 weeks of the study over 68% of subjects were compliant with their daily practice. When comparing baseline with week 2, the mean anxiety scores decreased from 25.4 to 16.8 (*p* < 0.01, *d* = 1.31). Similarly, mean depression scores decreased from 15 to 8.81 (*p* < 0.01, *d* = 0.9). The reduced scores for both anxiety and depression were maintained at weeks 4 and 6.

**Conclusion:**

This preliminary study assesses the effectiveness of online meditation training on self-reported symptoms of anxiety and depression. After 2 weeks of practice, those with baseline anxiety and depression showed significant improvement with a large effect size. The results from weeks 4 and 6 show sustained reduced anxiety and depression symptoms. These findings suggest that daily Isha Kriya practice could alleviate symptoms of these conditions. Future studies utilizing randomized control trials should be conducted to rigorously evaluate the benefits of this meditation practice on anxiety and depression.

**Trials registration:**

ClinicalTrials.gov, identifier: NCT05065476.

## Introduction

Anxiety and depression are some of the most prevalent ailments affecting a wide range of populations in the United States. In 2019, over 15% of the population experienced symptoms of anxiety with the highest reports amongst young adults aged 18–29-year. Among these adults, 9.5% experienced mild, 3.4% experienced moderate, and 2.7% experienced severe anxiety symptoms (1). The prevalence of anxiety and depression more than doubled during the pandemic and has continued to increase (2). In December 2020, 42% of those surveyed by the U.S. Census Bureau had symptoms associated with anxiety or depression compared to 11% of adults having similar symptoms from January to June 2019 (3). In addition, a recent report showed that symptoms associated with anxiety and depression increased between August 2020 to February 2021 from 36.4 to 41.5% in the U.S. (4).

Currently, treatments for anxiety and depression include pharmacotherapy with anxiolytics and antidepressants and/or psychotherapy. Between 2015 to 2018, the percentage of use of antidepressants over a 30-day period was reported to have increased with age; with higher consumption being reported in the elderly population (7.9% among those aged 18–39, 14.4% of those aged 40–59, to 19% of those aged 60 and over). In all age groups, women were seen to report higher antidepressant usage compared to men (5). In addition to medication such as antidepressants, Cognitive Behavioral Therapy (CBT) is a popular approach that is moderately effective on depression and anxiety. However, most of those with anxiety and depression do not receive treatment in part due to the lack of accessible assessment models and treatment for these mental illnesses in primary care settings. As a result, CBT is a non-scalable intervention in a population setting unless the primary care setup is better prepared for uniform delivery of CBT to the public and is equipped with adequate numbers of skilled mental health providers (6). In addition, individual CBT sessions can take close to an hour and require professional expertise, presenting a major time commitment for both patients and providers in primary care.

We propose a simpler, more scalable, and accessible intervention in the form of the Isha Kriya meditation. Isha Kriya is a meditation offered by Isha Foundation, a global non-profit organization which has conducted yoga and meditation programs for millions of people worldwide. Previous studies demonstrated positive effects of these programs on mental health (7–9), brain-derived neurotropic factor (BDNF) levels (7, 10), endocannabinoid levels (7), heart rate variability (HRV) (11), and brain functional connectivity (12). Isha Kriya is a simple 15-min practice which can be learned in a 1-h session by a trained instructor and the meditation itself is freely accessible online. A study demonstrated that a onetime experience of this guided meditation practice resulted in the reduction of stress and total mood disturbance amongst operating room professionals (13). This practice shows promise as a tool to improve mental health in a scalable manner and is an intervention worth exploring.

Mind-body interventions such as meditation have gained considerable popularity over the past decade. According to a CDC report, only 4.1% of U.S. adults reported meditating in 2012. By 2017, this number rose to 14.2% of U.S. adults—a greater than 3-fold increase in 5 years (14). The benefits of mind-body practices have been more closely studied in recent years. Meditation has been shown to improve emotional balance and attentiveness (15). A meta-analysis by Matko et al. (16) showed varying benefits of multicomponent mind-body practices with many studies demonstrating positive effects on anxiety and depression. Saeed et al.'s review evaluating the effects of exercise, yoga, tai chi, and mindfulness-based meditation on anxiety and depression showed that integrative yoga (based on meditation and breath control) was more effective than exercise-based yoga in reducing depressive symptoms (17). A review of pranayama (regulation of breath) by Jayawardena et al. reports that breath-based practices had both psychological and physiological benefits such as reduced anxiety among patients with cancer and cardiovascular diseases, improved cardio-respiratory functions among patients with bronchial asthma, and reduced systolic and diastolic blood pressure among patients with hypertension (18). Overall, meditation is effective in reducing symptoms associated with stress, anxiety, and depression (17, 19–21).

The COVID-19 pandemic has further revealed the importance of accessibility in healthcare and telemedicine. To reach a broader number of people, online delivery for meditation has been utilized for improving mental health and wellbeing (22). Isha Kriya has the potential as an additional tool to improve wellbeing due to its easy access, simplicity, and scalability. We hypothesize that the daily practice of Isha Kriya will improve states of anxiety and depression. In this study, we aim to accomplish the following:

- To assess the level of compliance with Isha Kriya over 6 weeks.
- To quantitatively assess the impact of the Isha Kriya practice on anxiety using a validated questionnaire over 6 weeks.
- To quantitatively assess the impact of the Isha Kriya practice on depression using a validated questionnaire over 6 weeks.

## Methods

### Subject recruitment

This observational study was designed to collect longitudinal data from subjects introduced to Isha Kriya meditation as an intervention and followed up for 6 weeks. Any individual who could read and understand English and showed an interest in completing the Isha Kriya webinars by enrolling in the course offered by the Isha Foundation, could participate in the study. The potential participant pool consisted of those from the public who chose to enroll for the webinar. Recruitment was conducted by sharing the study's survey link *via* advertisement emails to those who enrolled in the webinar, and participants self-reported on the eligibility criteria. Exclusion criteria included participants below 18 years of age and not U.S. residents. Study data were collected and managed using REDCap, an electronic data capture tool (23).

**Figure 1:**
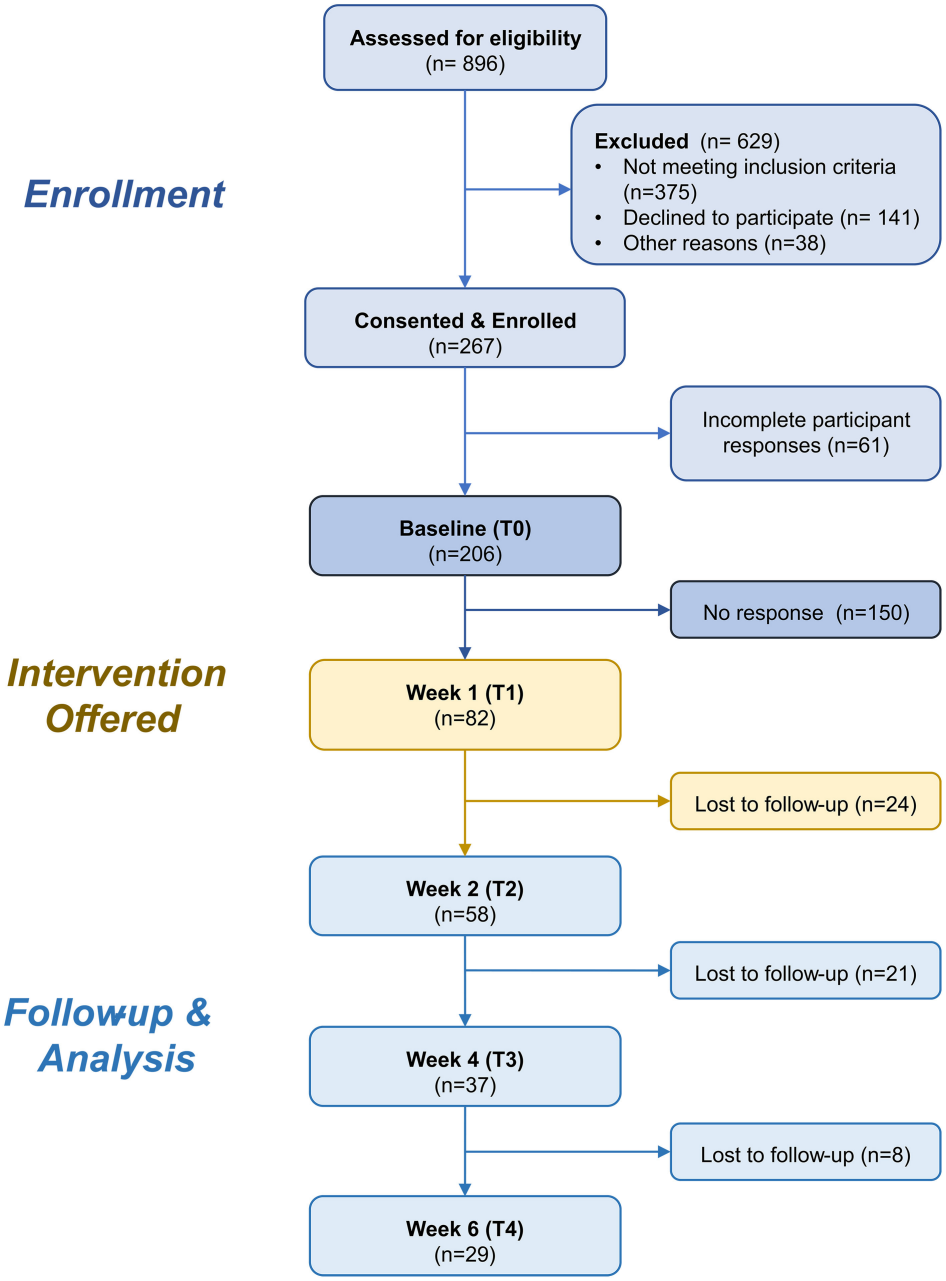
CONSORT Chart. Flowchart showing the number of participants at each stage of the study.

Upon consent, participants were asked to respond to the remotely administered survey at the following time points: at Baseline (T1), at week 2 (T2), at week 4 (T3), and finally at week 6 (T4). These surveys included validated questionnaires assessing anxiety (PROMIS) and depression scale (CESD-10). Self-reported meditation history and a 2-min, weekly compliance survey between T1 and T4 were also included. The compliance survey collected information on the frequency and duration of the prescribed meditation practices. The flowchart of participant numbers is shown in Figure 1.

### Intervention

Isha Kriya is a guided meditation practice that emphasizes centering thoughts with mindful breathing. A training video delivers step-by-step practice instructions. The 15-min practice aims to focus the mediator's attention beyond body and mind in three subsequent stages: (1) To combine mental affirmation of specific thoughts with the inhalation and exhalation, (2) to utter a given sound seven times, and (3) continue to sit in a seated posture with eyes closed. Anyone over 12 years of age can practice Isha Kriya. Participants were requested to practice Isha Kriya daily for the study duration.

### Outcome measures

#### Depression

Depression was measured by the Center for Epidemiological Studies-Depression (CES-D) 10-item scale (24, 25). The brief CES-D scale consists of 10 items assessing three factors; Depressed affect (blues, depressed, fear, lonely), Somatic retardation (bothered, sleep, get going, attention), and Positive affect (happy, hopeful). The response is coded from 0 (rarely) to 3 (most of the time) (26). The CESD-10 composite score is the sum of 10 scores. A value of 10 is considered the cutoff for depression. The CES-D scale has high reliability and validity and has remained a dominant measure for depression in the community population. This measure had strong reliability across study measurements, with α- 0.85 at baseline, α- 0.80 at 2 weeks, α- 0.78 at 4 weeks and α- 0.77 at 6 weeks.

#### Anxiety

Anxiety was measured by the 8-item Patient-Reported Outcomes Measurement Information System (PROMIS) Emotional Distress—Anxiety (Short Form) (27). The response is coded on a 5-point scale from 1 (never) to 5 (always). The PROMIS-Anxiety item banks assess self-reported fear (fearfulness, panic), anxious misery (worry, dread), hyperarousal (tension, nervousness, restlessness), and somatic symptoms related to arousal (racing heart, dizziness). The PROMIS Anxiety composite score is the sum of the eight scores. Composite scores between 17 and 21 are considered mild anxiety, scores between 22 and 31 are considered medium anxiety, and scores above 31 are considered severe anxiety. The anxiety measures are universal rather than disease-specific and assess anxiety over the past 7 days. This measure had strong reliability across study measurements, with α- 0.96 at baseline, α- 0.94 at 2 weeks, α- 0.93 at 4 weeks, and α- 0.96 at 6 weeks.

#### Compliance

Compliance was defined as 60% of activity completed during the intervention period. This value was selected based on prior studies (28–30). This information is obtained *via* the self-reported weekly compliance diaries collected from the participants.

### Statistical analysis

#### Compliance/retention

Baseline characteristics of participants were recorded by calculation of frequency and proportions (%). Participants were categorized based on cutoff values to assess if changes were different based severity of reported symptoms. Baseline participants were split into tertiles (A1: High, A2: Medium, A3: Low) based on baseline anxiety. We also divided it into another tertile (D1: High, D2: Medium, D3: Low) based on baseline depression scores. To investigate the anxiety and depression scores between retained and dropped participants, we conducted multiple linear regression on outcomes adjusted with age, gender, and previous mediation experience.

#### Survey results

Anxiety and Depression scores were recorded with means (SD) and medians (IQR) on each depression/anxiety tertile group for all timepoint. Linear mixed effect models were conducted using time, age, gender, and meditation experience as fixed effect and subjects as a random effect to account for the multiple measurements per subject for PROMIS and CESD-10 scores. The sub-analyses were performed in linear mixed effect models using different cutoffs for splitting anxiety/depression groups. An unstructured covariance matrix was used for the random effects as the variance-covariance structure. Time (Baseline, Weeks 2, 4, and 6) and age (18–29, 30–44, 45–64, 65+) each have four levels at which the data was measured. Gender (male/female), and past meditation experience (yes/no) both have two levels.

The initial cutoffs for the anxiety scores were based on the recorded PROMIS score at the baseline that is Tertile A1 (28<), Tertile A2 (22<28), and Tertile A3 (<21). We also performed additional analysis using clinical severity cutoff values based on the baseline scores to better understand the potential clinical implications of this intervention. The clinical severity categories are <17 none, 18–21 mild, 22–31 moderate, and 32 < severe anxiety.

The cutoffs for the depression groups were based on the recorded CESD-10 score at the baseline, that are Tertile D1 (19–33), Tertile D2 (10–18), and Tertile D3 (3–9). Similarly, we performed additional analysis using clinical cutoff values based on the baseline scores. The clinical categories for the CES-D scale are < 10, no depression, and 10 ≤ depression.

All statistical analyses were conducted using the R statistical software (The R Foundation for Statistical Computing, Vienna, Austria, version 4.1.2) with a two-sided *p*-value < 0.05 considered statistically significant. Effect size was calculated using Cohen's d with values 0.2–0.49 considered small, 0.5–0.79 considered medium, and 0.8 and above considered large.

## Results

### Subject characteristics

Table 1 describes the baseline characteristics of the 267 subjects who met the eligibility criteria and were enrolled in the study. The majority of these subjects were in the 45–64 age group (44%), followed by the 30–44 age group (34%). Seventy-six percent of the subjects were identified as female. More participants had past meditation experience than those without any meditation experience (53 vs. 44%).

**Table 1:** Baseline characteristics of all participants initially enrolled in the study.

	**Enrolled *n* = 267**
**Age**
18–29	26 (10%)
30–44	90 (34%)
45–64	117 (44%)
64+	27 (10%)
n/a	7 (3%)
**Gender**
Male	57 (21%)
Female	202 (76%)
Other	1 (0.3%)
n/a	7 (3%)
**Past meditation experience**
Yes	142 (53%)
No	118 (44%)
n/a	7 (3%)

Out of the initial 267 enrolled participants, 82 completed a week 1 compliance survey which confirmed their participation in the intervention webinar and demonstrated continued interest in the study. Since the webinars were conducted by Isha Foundation and the study team had no access to the information on participants' webinar attendance, which was a proxy for their meditation training, the study team relied heavily on participants' self-reported webinar check questionnaire responses. Hence, data from these 82 confirmed participants was used as the baseline data for analysis. We believe this approach enables greater specificity of the data reported and reduces the chance of incorrect interpretations of the results.

Table 2 shows the baseline characteristics of the 185 participants who dropped out before week 1 and the 82 confirmed participants we call “baseline” going forward. The baseline group had 12% fewer subjects below the age of 44 and 15% more subjects above 64. This group also had 11% more female subjects and 18% more subjects with meditation experience. Multiple linear regressions were performed for PROMIS Anxiety and CESD-10 Depression scores comparing dropped vs. confirmed baseline participants adjusted with age, gender, and past mediation experience. The regression for PROMIS scores indicated a significant difference (*p* = 0.04) in average baseline PROMIS scores, 1.2 points higher for baseline participants than the dropped participants adjusted with age, gender, and past meditation experience. Using the same adjustments (age, sex, and past meditation experience), we did not observe a significant difference in CESD-10 scores between the two groups at the 0.05 level (*p* = 0.51).

**Table 2:** The table shows the characteristics of dropped and baseline participants.

	**Dropped**	**Baseline**	***p*-value**	**Cohen's**
	**(*n* = 185)**	**(*n* = 82)**		***d***
**Age** ***n***
18–29	21(11%)	5 (6%)		
30–44	66 (36%)	24 (29%)		
45–64	79 (43%)	38 (46%)		
64+	12 (6%)	15 (18%)		
n/a	7 (4%)			
**Gender**
Male	43 (23%)	14 (17%)		
Female	134 (72%)	68 (83%)		
Other	1 (1%)			
N/A	7 (4%)			
**Past meditation experience**
Yes	88 (48%)	54 (66%)		
No	90 (49%)	28 (34%)		
N/A	7 (4%)			
**PROMIS**
Mean ± S.D.	24.2 ± 6.53	25.4 ± 7.61	0.04	0.16
Median (IQR)	25 (8)	25 (10)		
**CESD-10**
Mean ± S.D.	14.9 ± 7.32	15.1 ± 7.65	0.51	0.03
Median (IQR)	15 (10.75)	14.5 (12)		

*P*-values are from the multiple linear regressions comparing dropped and baseline participants adjusted with age, gender, and past mediation experience.

### Compliance

**Figure 2:**
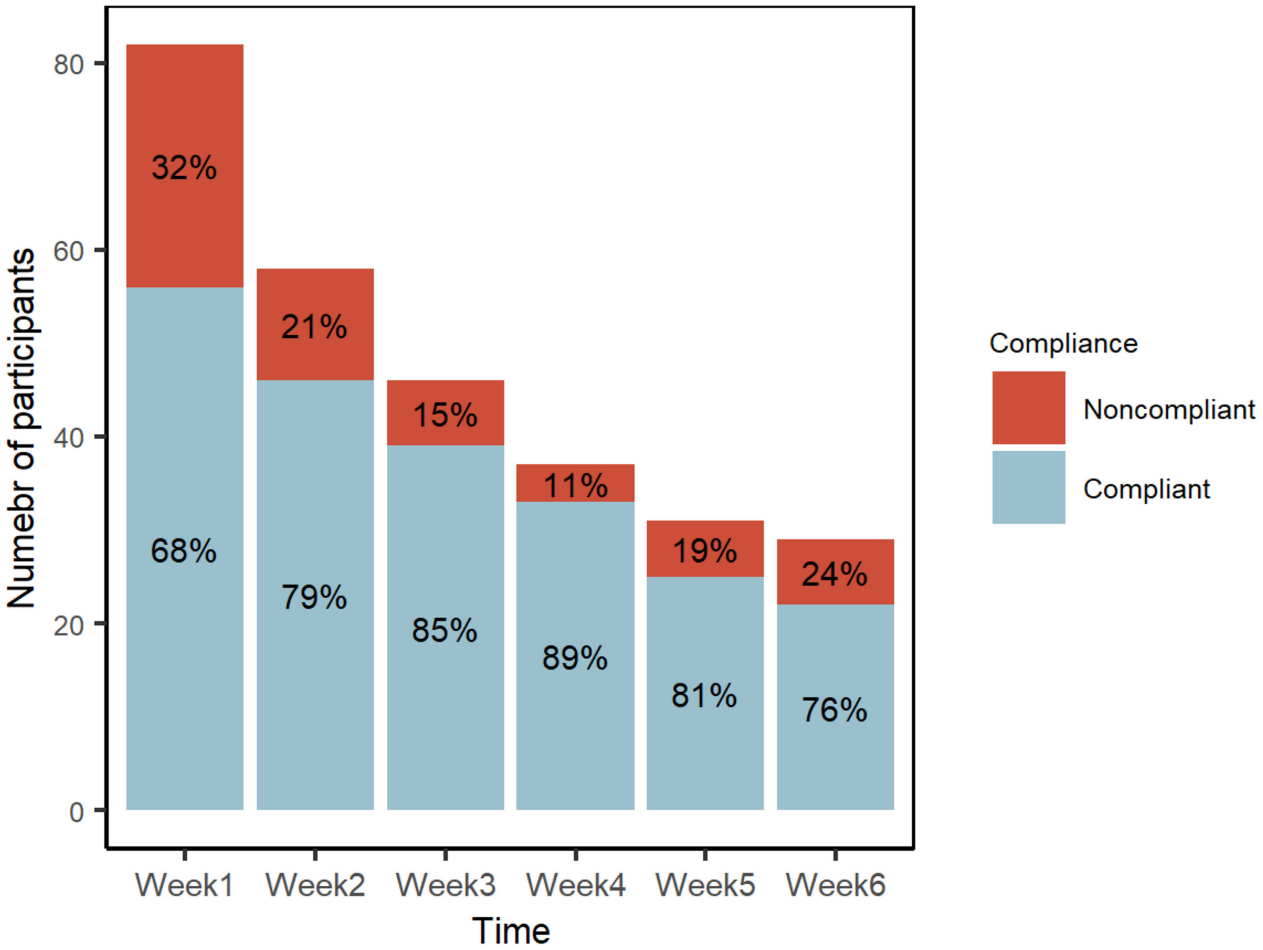
Proportions of compliant and non-compliant participants over the study period.

Compliance was defined as 60% completion of the study activities, namely performing the practice at least once a day for 4 days a week or more. Figure 2 shows the ratio of compliant vs. non-compliant participants at each timepoint from weeks 1 to 6, with 68 to 89% compliance. When comparing the baseline with week 6, we observed a large drop out of the younger participants (100% attrition in the 18–29 age group, 63% attrition in the 30–44 age group). In the study, we also observed that the retention was higher in those who have past meditation experience than those without experience (39 vs. 29% at week 6) (Supplementary Table A1).

### Normality of scales

To evaluate anxiety and depression, we used a linear mixed effect model to assess statistically significant differences in scores. The normality of PROMIS was assessed by the Shapiro-Wilk test (T1: *p* = 0.19, T2: 0.23, T3:0.05, and T4: 0.06), indicating normal distribution for each timepoint. The normality of CESD-10 score were assessed by the Shapiro-Wilk test (T1: *p* < 0.01, T2: *p* < 0.01, T3: *p* = 0.02, T4: *p* = 0.02) indicating non-normal distributions for each timepoint.

### Effect of control variables

We assessed the effect age, gender, and prior meditation experience on anxiety and depression scores. None of these variables had a significant impact on anxiety scores. On the other hand, prior meditation experience had significant impact (*p* = 0.02) on depression outcomes. Those with prior meditation experience had significantly lower depression scores overall compared to those without. The data is summarized in Tables 3, 4.

**Table 3:** Mixed effect model estimates, *p*-value, type 3 *p*-value for PROMIS scores.

	**Estimate**	**95% C.I**.	*p*-value*	Type 3 *p*-value**
**Time**	
Baseline	-	-	-	< 0.01
Week 2	−8.49	(−10.1, −6.86)	< 0.01	
Week 4	−9.70	(−11.79, −7.60)	< 0.01	
Week 6	−9.24	(−11.55, −6.94)	< 0.01	
**Age**	
18–29	2.24	(−3.89, 8.36)	0.47	0.35
30–44	3.59	(−0.35, 7.52)	0.07	
45–64	2.02	(−1.60, 5.64)	0.27	
65+	-		-	
**Gender**	
Female	−1.80	(−5.22, 1.62)	0.30	0.30
Male	-	-	-	
**Meditation experience**			
Yes	−2.30	(−5.02, 0.41)	0.10	0.10
No	-	-	-	

From the mixed effect model, we observed the PROMIS score decreased by 9.24 points on average from baseline to week 6 after adjusted with age, gender, and meditation experience. Particularly, we observed a significant change of PROMIS score at week 2 (8.49, p < 0.01) from the baseline. ^*^Based on Satterthwaite's method.

^**^Based on F-tests, based on Satterthwaite's method.

**Table 4:** From the mixed effect model, we observed the CES-D-10 score decreased 6.16 points on average from baseline to week 6 after adjusting with age, gender, and meditation experience (*p* < 0.01).

	**Estimate**	**95% C.I**.	*p*-value*	Type 3 *p*-value**
**Time**
Baseline	-	-	-	< 0.01
Week 2	−6.09	(−7.8, −4.4)	< 0.01	
Week 4	−6.00	(−8.3, −3.7)	< 0.01	
Week 6	−6.16	(−8.3, −4.1)	< 0.01	
**Age**
18–29	2.83	(−2.9, 8.5)	0.30	0.36
30–44	2.95	(−0.7, 6.6)	0.07	
45–64	1.10	(−2.3, 4.5)	0.25	
65+	-		-	
**Gender**
Female	−0.99	(−4.1, 2.2)	0.68	0.53
Male	-	-	-	
**Meditation experience**		
Yes	−3.11	(−5.6, −0.6)	0.02	0.02
No	-	-	-	

At week 2, we observed the largest change of score (6.09). We also observed those who had past mediation experience showed a significant reduction of 3.11 (p = 0.02) on CES-D 10 score compared to those without past meditation experience.

^*^Based on Satterthwaite's method.

^**^Based on F-tests, based on Satterthwaite's method.

### Reduced anxiety in all tertiles

Table 5 depicts the mean and median anxiety scores at each time point and the p values and Cohen's d when compared to the baseline for all study participants (overall) and each tertile (A1, A2, and A3). The linear mixed effect model revealed statistically significant differences in anxiety scores at weeks 2, 4, and 6 compared to the baseline (*p* < 0.01) for the overall data as well as the high (A1) and medium (A2) tertiles but no significant change in the low tertile (A3). Compared to the baseline, Cohen's d showed large effect sizes, especially in the 2nd week (overall *d* = 1.31, tertiles *d* = 0.84–2.25).

**Table 5:** Table shows the anxiety scores for both compliant and non-compliant participants for each tertile group.

	**Baseline**	**Week 2**	**Cohen's d**	**Week 4**	**Cohen's d**	**Week 6**	**Cohen's d**
			***p*-value**		***p*-value**		***p*-value**
**Overall**	
Mean (SD)	25.4 (7.61)	16.8 (6.24)	1.31	16.0 (6.04)	1.30	16.3 (6.71)	1.22
Median (IQR)	25 (10)	16 (9)	* < 0.01	15 (8.5)	* < 0.01	16 (9)	* < 0.01
*N*	82	58		37		28	
**Tertile A1**	
Mean (SD)	33.1 (3.30)	19.9 (7.17)	2.25	17.7 (7.96)	2.67	19.9 (9.31)	1.37
Median (IQR)	32 (4.5)	20 (10)	< 0.01**	20 (12.5)	< 0.01**	20.5 (15)	< 0.01**
*N*	31	22		12		9	
**Tertile A2**	
Mean (SD)	24.4 (2.19)	17.2 (4.34)	1.48	16.4 (4.73)	1.39	16.6 (3.96)	1.68
Median (IQR)	24 (4.75)	17 (6.75)	< 0.01**	16 (7)	< 0.01**	16 (6)	< 0.01**
*N*	26	20		14		13	
**Tertile A3**	
Mean (SD)	16.7 (4.53)	12.5 (4.76)	0.84	13.3 (4.73)	1.12	12.3 (5.82)	1.15
Median (IQR)	18 (8)	12 (5)	0.15**	12.0 (5.5)	0.67**	10.5 (5)	0.47**
*N*	25	16		11		8	

Effect sizes, Cohen's d for paired sample, were computed by comparing baseline values and other timepoint values. *P*-values are computed by repeated measure analysis using linear mixed effect models.

^*^p-value by linear mixed effect model.

^**^p-value by stratified linear mixed effect models.

**Figure 3:**
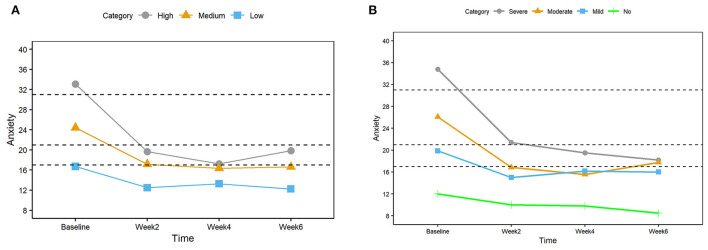
**(A)** Mean anxiety scores for three groups (High, Medium, and Low) based on the tertiles at the baseline. The dashed horizontal lines indicate clinically significant cutoffs (17, 21, and 31). **(B)** Mean anxiety scores for four groups (Severe, Moderate, Mild, and No) based on the clinically significant cutoffs at baseline.

Figure 3A shows the mean anxiety scores at each study time point for the high (gray), medium (orange), and low tertiles (blue). For all tertiles, the biggest reduction in scores happened between baseline and week 2, and the lower scores were maintained for the next 4 weeks of the study.

### Reduced anxiety in all clinical groups

We examined the anxiety scores of four clinically significant cutoff groups (<17 none, 18–21 mild, 22–31 moderate, >32 severe) to observe their trend during the study period. We observed large effect sizes ranging from 0.76 to 6.36 when comparing weeks 2, 4, and 6 with the baseline for all four clinical severity categories (Table 6).

**Table 6:** The table shows the mean and medians of anxiety scores of overall and four groups based on clinically significant cutoffs.

	**Baseline**	**Week 2**	**Cohen_d**	**Week 4**	**Cohen_d**	**Week 6**	**Cohen_d**
			***p*-value**		***p*-value**		***p*-value**
Overall							
Mean (SD)	25.4 (7.61)	16.8 (6.30)	1.31	16.0 (6.04)	1.30	16.3 (6.71)	1.22
Median (IQR)	25 (10)	16 (9)	* < 0.01	15 (8.5)	* < 0.01	16 (9)	* < 0.01
N	82	58		37		28	
Severe			2.38		2.63		1.55
Mean (SD)	34.8 (2.86)	21.4 (7.30)	-	19.5 (6.97)	-	18.2 (9.86)	-
Median (IQR)	34 (5)	23 (10)		21.5 (10.2)		18 (17)	
*N*	20	15		7		5	
Moderate			1.22		1.13		1.34
Mean (SD)	26.1 (3.20)	16.9 (4.49)	-	15.6 (5.61)	-	17.8 (5.51)	-
Median (IQR)	26 (6)	17 (8)		14 (7)		16 (7)	
*N*	37	27		18		16	
Mild			2.51		6.36		4.95
Mean (SD)	19.9 (1.46)	15 (5.37)	-	16.2 (4.54)	-	16 (6.38)	-
Median (IQR)	20 (1.5)	15 (4.75)		15.5 (4)		14 (6.5)	
*N*	15	8		6		4	
No			1.29		0.76		1.65
Mean (SD)	12 (3.20)	10 (2.33)	-	9.8 (1.64)	-	8.5 (1)	-
Median (IQR)	11.5 (6)	9.5 (2.75)		9 (2)		8 (0.5)	
N	10	8		5		4	

Effect sizes, Cohen's d for paired sample, were computed by comparing baseline values and other timepoint values. *P*-values are computed by repeated measure analysis using linear mixed effect models.

^*^p-value by linear mixed effect model.

As depicted in Figure 3B, results were in agreement with that of the tertile approach, with the largest reduction of anxiety scores in the 2nd week and sustained low scores afterward. As seen in Figure 3 the highest tertile (A1) and the severe clinical category showed the largest reduction compared to the lower anxiety score groups. The severe, moderate, and mild groups ended the study at a score near or below the clinical cutoff score of 17.

### Reduced anxiety in completed-only group

**Figure 4:**
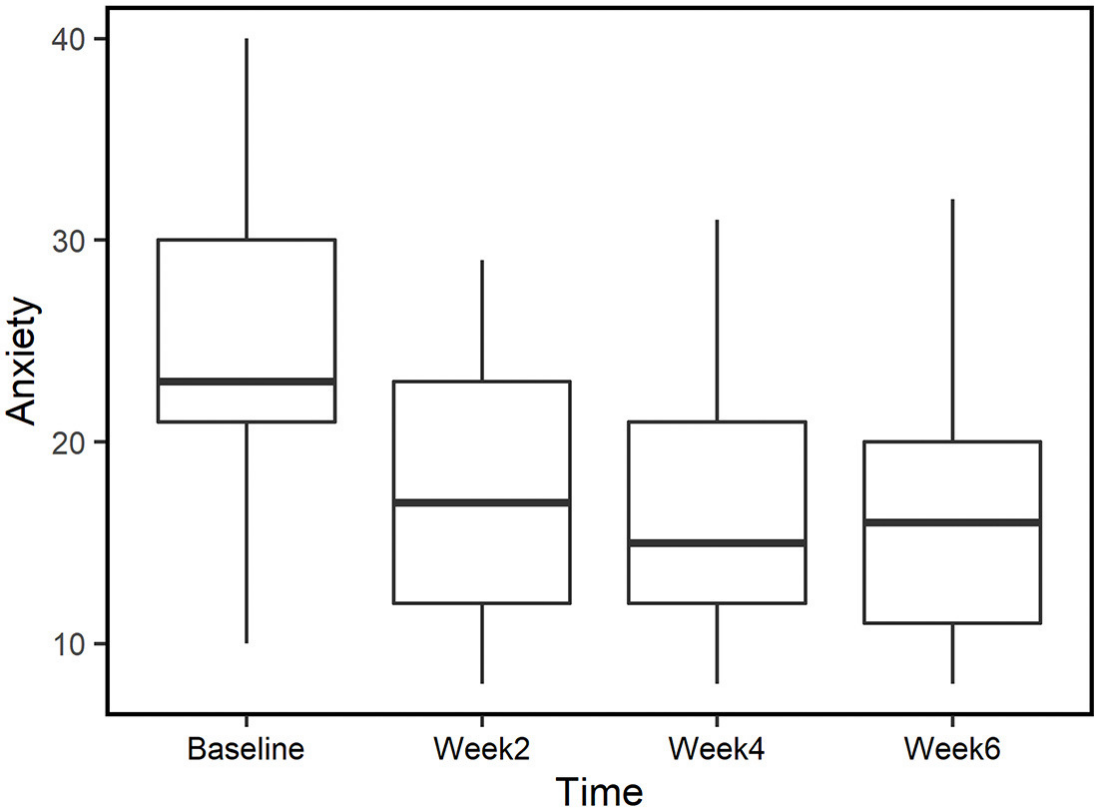
Anxiety scores for those who completed all time points (*n* = 29). Median values are represented by horizontal black lines in the box plot.

To ensure that the results are not biased by the different number of subjects at each time point, we also examined anxiety score for those participants who completed all time points (*n* = 29). The trend in this group is similar to that of the complete data set (Tables 3, 4). Mean anxiety score was significantly reduced by week 2 (*P* < 0.01, *d* = 1.19) and the effects were sustained at week 4 (*P* < 0.01, *d* = 1.22) and week 6 (*P* < 0.01, *d* = 1.22), showing large effect sizes at all time points when compared to baseline (Figure 4; please refer to Supplementary Table A2 for further detail).

### Reduced depression in all tertiles

Table 7 shows the mean and median depression scores along with p-values and Cohen's d for each study time point compared to the baseline for the overall data and tertile categories (D1 high, D2 medium, D3 low). From the linear mixed effect model, we observed the statistically significant (*p* < 0.01) differences between the baseline mean of depression score and means of all other time points in the overall data and the high (D1) and medium (D2) tertiles. The changes were not statistically significant for the low tertile (D3). Cohen's d showed a large effect size, especially in the 2nd week (*d* = 0.90) and high depression tertile (D1, *d* = 2.66).

**Table 7:** The table shows the mean and medians of depression scores of overall and tertile groups.

	**Baseline**	**Week 2**	**Cohen_d**	**Week 4**	**Cohen_d**	**Week 6**	**Cohen_d**
			***p*-value**		***p*-value**		***p*-value**
Overall							
Mean (SD)	15 (7.42)	8.81 (5.69)	0.90	8.58 (5.70)	0.74	8.14 (5.50)	0.85
Median (IQR)	15 (12)	7.5 (7.75)	< 0.01*	7 (8)	< 0.01*	6 (6)	< 0.01*
*N*		58		37		28	
**Tertile D1**	
Mean (SD)	23.6 (3.7)	11.4 (6.0)	2.66	11.3 (6.6)	1.92	11.7 (5.7)	1.79
Median (IQR)	22.5 (6.5)	12 (8)	< 0.01**	11 (10.25)	< 0.01**	11 (6)	< 0.01**
*N*	30	21		10		8	
**Tertile D2**	
Mean (SD)	13.6 (2.8)	10.3 (5.9)	0.52	8.25 (5.5)	0.94	7.9 (5.2)	1.05
Median (IQR)	13 (5)	10 (7)	< 0.01**	8 (5.25)	< 0.01**	6.5 (5.8)	< 0.01**
*N*	26	16		12		10	
**Tertile D3**	
Mean (SD)	6.77 (2.0)	5.2 (2.8)	0.47	7.71 (6.3)	0.08	5.91 (5.2)	0.32
Median (IQR)	7 (3.5)	5 (4)	0.09**	5 (4.75)	0.78**	5 (1)	0.24**
*N*	26	20		14		11	

Effect sizes, Cohen's d for paired sample, were computed by comparing baseline values and other timepoint values. *P*-values are computed by repeated measure analysis using linear mixed effect models.

^*^p-value by linear mixed effect model.

^**^p-value by stratified linear mixed effect models.

**Figure 5:**
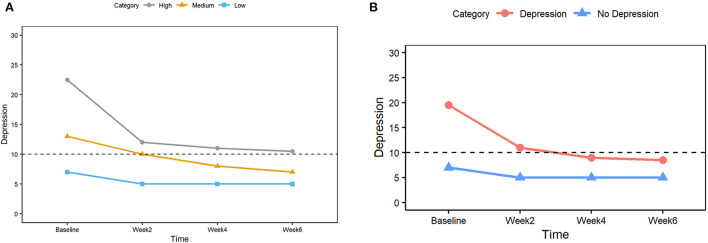
**(A)** Median depression scores for three groups (High, Medium, and Low) based on the tertiles at the baseline. The dashed horizontal line indicates the clinically significant cutoff (score 10 or higher considered to be depression). **(B)** Median depression scores for two groups (Depression, No depression) based on the clinically significant cutoff of 10 at the baseline. The dashed horizontal line indicates the clinically significant cutoff (score 10 or higher considered to be depression).

Figure 5A shows the median values of the CES-D depression score at each study timepoint for each tertile category. The largest change is observed between baseline and week 2 in all 3 tertile categories, and the reduced scores were maintained thereafter. Comparing baseline and week 6, the high tertile score medians were reduced from 22.5 to 11, and medium tertile score medians were reduced from 13 to 6.5. A score of 10 (depicted by a horizontal dashed line) is considered a depression threshold for the CES-D score.

### Reduced depression in all clinical groups

To assess the impact of this intervention from a clinical perspective, the subjects were categorized into two groups based on their baseline depression scores: the depression group for baseline scores above and including 10 and the no-depression group for baseline scores below 10. The depression group showed a large effect size compared to the baseline at all time points. The results were in agreement with that of the tertile approach, showing the largest change between baseline and week 2 and sustained lower scores thereafter. As detailed in Table 8, depression scores were significantly lower (*p* < 0.01) at all timepoints compared to the baseline for both depression and no-depression groups.

**Table 8:** The table shows the depression scores of two groups based on the clinically significant score cutoff of 10 for all time points.

	**Baseline**	**Week 2**	**Cohen's d**	**Week 4**	**Cohen's d**	**Week 6**	**Cohen's d**
			***p*-value**		***p*-value**		***p*-value**
**Depression**	
Mean (SD)	19 (6.03)	10.9 (5.90)	1.20	9.64 (6.10)	1.25	9.5 (5.40)	1.28
Median (IQR)	19.5 (9.5)	11 (8)	< 0.01	9 (7.5)	< 0.01	8.5 (8)	< 0.01
*N*	56	37		22		18	
**No depression**	
Mean (SD)	6.77 (2.03)	5.2 (2.80)	0.47	6.93 (4.76)	0.08	5.91 (5.13)	0.32
Median (IQR)	7 (3.5)	5 (4)	< 0.01	5 (4.75)	< 0.01	5 (1)	< 0.01
*N*	26	20		14		11	

Figure 5B shows the median CES-D scores for the depression (red) and no-depression (blue) groups at all study time points, with the horizontal dashed line signifying a threshold score of 10. The depression group started with a high score of 19.5 at baseline and completed the study at a low score of 8.5 (below the threshold score of 10).

### Reduced depression in completed-only group

**Figure 6:**
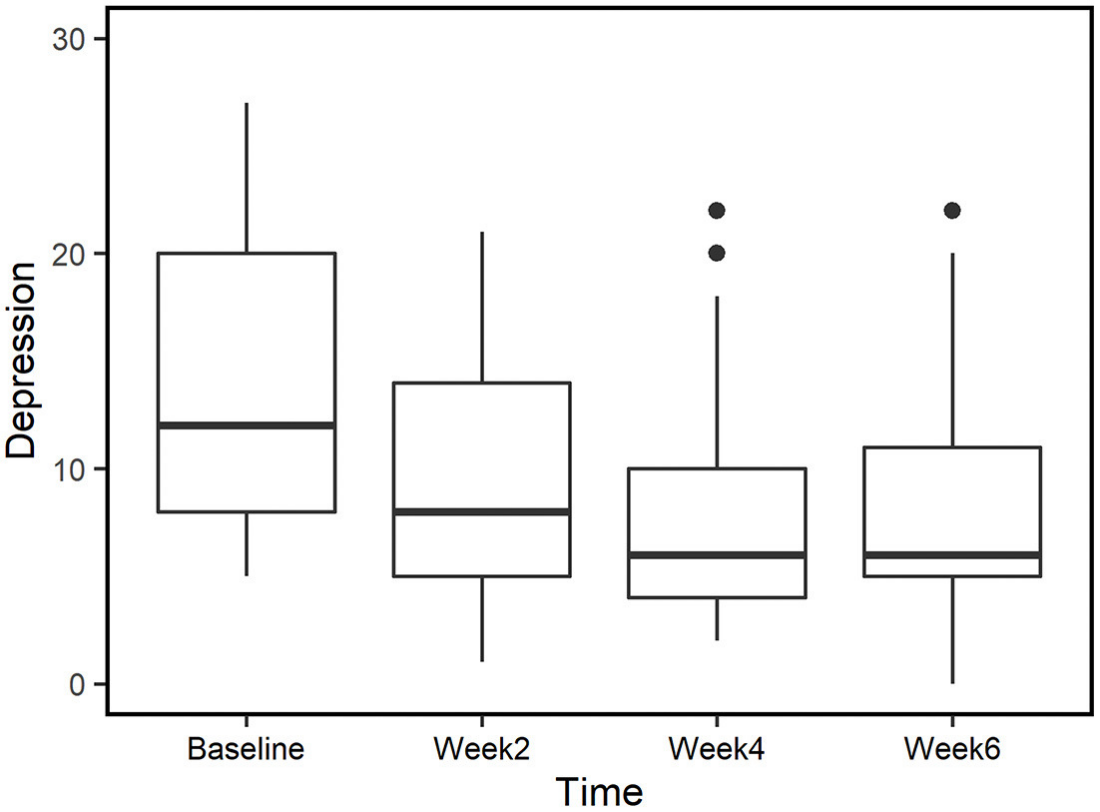
Depression scores for those who completed all time points (*n* = 29). Median values are represented by horizontal black lines in the box plot.

Similar to our approach to evaluate changes in anxiety, we examined depression score for those participants who completed all time points (*n* = 29). The trend in this group is similar to that of the complete data set (Tables 5, 6). Mean depression score was significantly reduced by week 2 (*p* < 0.01, *d* = 0.66) and the effects were sustained at weeks 4 (*p* < 0.01, *d* = 0.72) and 6 (*p* < 0.01, *d* = 0.85), showing moderate to large effect sizes when compared to baseline (Figure 6; Please refer to Supplementary Table A3 for further detail).

## Discussion

This observational pilot study evaluates the impact of online meditation training on self-reported symptoms of anxiety and depression. Participants with baseline anxiety and depression showed significant improvement (*p* < 0.01) in both conditions with a large effect size (*d* > 0.8). Improvements were seen as early as 2 weeks from the conveyance of the intervention and were sustained over 6 weeks. Although study participation declined as the investigation progressed, most of those who continued with the study remained compliant with their practices. For those who completed all time points, trends in improvement for anxiety and depression were similar to the larger group that included all participants at each time point.

Subjects with severe to medium anxiety scores at baseline reported a decline in their symptoms to mild anxiety, and those with mild anxiety were able to report levels associated with no anxiety. Similar improvements were recorded in depression symptoms. The median scores for subjects who initially reported symptoms of depression saw values decline to below the cutoff for depression after 4 weeks of Isha Kriya practice. Participants whose scores belonged in the highest tertile at the beginning of the study (A1/D1) demonstrated the largest reductions in the scores compared to participants who had lower scores. Reduction of reported symptoms of anxiety and depression after meditation are consistent with those found in the literature (31–35).

While findings of reduced anxiety and depression following meditation were consistent with prior studies, the overall magnitude of the effect in this study is notable. A meta-analysis of various positive psychological interventions by van Agteren et al. showed that approaches such as mindfulness were effective in improving mental wellbeing in both clinical and non-clinical populations (36). However, the most optimistic results were of moderate effect size. The participants in our study reported improvements in anxiety and depression levels that were of large effect size after 2 weeks of meditation practice and these improvements were maintained for the rest of the study. It is important to note that this comparison/analysis was done in the intervention group in the absence of controls. A majority of those who started depressed were reporting no longer experiencing clinical levels of depression by the study's end. Collectively, these findings suggest that meditation, even with brief online delivery, can be a powerful tool in alleviating symptoms of anxiety and depression.

The efficacy of mindfulness practices in improving mental health outcomes has been extensively reviewed in recent times. With the advent of the COVID-19 pandemic, measures of social distancing and work from home were introduced. These measures resulted in a greater number of people staying indoors and leading a more sedentary lifestyle with accentuated feelings of isolation (37). Moreover, reports of psychological distress (38, 39), anxiety (40), sleep disturbances (41), and problematic substance use have increased (42). Several studies were conducted to explore the feasibility of employing remotely delivered intervention that might attenuate these symptoms. Yoga and meditation approaches have been shown to improve mental health and wellbeing in the past. Online delivery of meditation has shown to be effective in improving mental health outcomes (43, 44); especially for anxiety symptom relief during the COVID-19 pandemic (45).

A recently published randomized control trial demonstrated online yoga interventions had a beneficial effect on student's mental health with 12 weeks of yoga practice (30). Studies on Inner Engineering Online (IEO), a multicomponent self-paced online program with lessons illustrating meditation and yoga practices, have been shown to reduce stress (29) and improves subjective wellbeing (46). Upon completion of IEO, participants can sign up to learn the Shambhavi Mahamudra Kriya (SMK) practice. Regular practice of SMK has been shown to reduce perceived stress and improve general wellbeing (47). Yet another study successfully demonstrated that online initiation for SMK followed by routine practice resulted in improved mental health outcomes (28). These studies agree with our assertion that web-based intervention delivery is, in fact, a feasible approach in the present day and age.

Compliance is a confounding yet compelling aspect of this study. Compliance was defined as 60% of activity completed during the intervention period. Initially, the study protocol dictated that all participants should practice Isha Kriya meditation twice daily for 6 weeks. However, the study protocol was revised to adopt a more pragmatic definition of compliance as a minimum of 4 days a week of Isha Kriya practice at a minimum practice frequency of once a day. The study participants who confirmed attending the introductory webinar demonstrated over 68% compliance to Isha Kriya practice during the study period. The rate of participant dropout ranged between 11–32% at each timepoint for these participants, which amounts to an aggregate of 64.6% dropout rate from weeks 1 to 6 (Figure 2). These figures are in line with the other mindfulness studies with attrition rates ranging from 8 to 60% (48, 49).

One of the main reasons behind this high dropout rate is likely the lack of any support mechanism to help participants learn and incorporate the new practice into their daily routine. Since this was an observational study, the study team did not incorporate compensation or any follow up mechanism for those who missed the surveys or had incomplete responses. Another reason could be the fact that the webinar was offered free of charge. Although this is an important factor for scalability, it may cause the meditation to be perceived as less beneficial than more expensive treatments. We observed a statistically significant difference (*p* = 0.04) in the baseline anxiety scores between the early dropouts and confirmed baseline participants (Table 2). However, the calculated effect size of 0.16 is below the threshold of 0.2 for a small effect size. This suggests that though the difference is statistically significant, the difference in effect is negligible. Finally, the dropout group in this study did not demonstrate any statistical differences in anxiety (Supplementary Table A4) or depression (Supplementary Table A5) at any of the time points. This suggests that dropouts were most likely not associated with the effect of the meditation.

Consistent practice is often recognized as a key factor in experiencing the positive impact of mind-body interventions. In a meta-analysis of mindfulness, performed by Cavanagh et al. (50), 5 out of 15 studies established a dose-response relationship between compliance as an engagement metric and study outcomes. They further commented that studies reporting greater engagement also noted an increased correlation between intervention engagement, completion of sessions or higher intervention practice, and improved study outcomes.

Regular practice of Sudharshan Kriya has been shown to alleviate symptoms associated with anxiety disorders and depression (51–53). Furthermore, it has shown to be effective in treating depression in those who have previously been resistant to antidepressant therapy (54). The practice of Transcendental Meditation (55, 56) and mindfulness-based stress reduction (57–59) has also been shown to offer noticeable improvements in mental health. Another study observed the impact of implementing an online meditation program for medical students (60). While there was some interest, there was poor compliance with limited reported benefits. Our study shows that compliance is a significant challenge for most remotely delivered meditation programs (43, 60). For motivated participants, online meditation offers a convenient and scalable opportunity for self-improvement. However, in participants who lack motivation, compliance with practices could be a major challenge (43).

Overall, this study demonstrated the promise of an online meditation intervention, Isha Kriya, for those with anxiety and depression. Those who were compliant with the meditative practice reported significant improvements in their conditions. As this was a preliminary study, the authors recommend future studies incorporating more rigorous protocols, such as using randomized control trials, to evaluate the effects of Isha Kriya practice on mental health outcomes.

### Strengths and limitations

Based on the findings of this study, insightful conclusions can be substantiated about the impact of Isha Kriya on symptoms of depression and anxiety. The initial recruitment numbers suggest a considerable interest in the masses to utilize web-based yoga and meditation interventions to alleviate symptoms of anxiety and depression. The high ratio of compliant vs. non-compliant subjects at each timepoint suggests feasibility and acceptability of this intervention in the general population.

The significant decline in anxiety and depression scores of participants with high clinical severity (medium to severe) at the beginning of the study to subclinical or near subclinical scores, with just 2 weeks of meditation practice suggests that this intervention may be a candidate for an effective adjunct therapy option for the clinical populations with depression and anxiety. Many depressed participants demonstrated scores below the threshold after 2 weeks with sustained effects for up to 6 weeks.

Being an online free meditation available in 13 different languages makes this meditation uniquely suitable as a scalable intervention that can easily be made available to a large audience worldwide, especially the underserved and minority communities. The compelling findings of this project call for more rigorous studies on this intervention to address the growing need for accessible telehealth tools to alleviate the current mental health crisis. This is essential in our collective effort to reduce healthcare disparities.

This study has several limitations. Since we chose an observational study design, control group was not included in the study. Hence, the authors are unable to account for a perceived 'placebo effect' from the intervention. Study participants could acutely be aware of the perceived beneficial effects of meditation practice resulting in a false sense of symptom alleviation. This effect is further compounded by the fact that study participants were recruited from a pool of individuals who had completed registration formalities to enroll in the free webinars offered by the Isha Foundation. This introduces selection bias to the study. Unfortunately, even by employing the best of study designs, i.e., randomized controlled trials, the element of selection bias in mindfulness-based studies cannot be completely mitigated (61). However, to accommodate this bias the study team ensured rigorous analysis of the data collected. The data was analyzed in multiple computations, and linear mixed effect model was employed to account for both known & unknown confounders. Large attrition rate is one of the limitations in many meditation studies. Despite the lack of methods to boost retention and adherence (to reflect real world lack of nurturing personnel for online, guided, meditation methods, the 70% compliance rate is consistent with the reported literature. Potential approaches for future studies would include incorporating financial compensation for study participation, and regular follow-ups with those who do not respond to the study surveys. In the current study, the majority of participants were compliant at each timepoint. As a result, due to the small sample size in the non-compliant group, the study team was not able to perform statistical analysis to delineate the possible dose response associated with compliance. Sample sizes of other subgroups based on symptom severity were also small, hence reducing the reliability of these reported values. Future studies with larger sample size are needed to address these limitations.

Moreover, as previously mentioned, data was not collected beyond 6 weeks. Therefore, the long-term implications of daily practice of this meditation on mental health are unknown. Finally, detailed knowledge of demographic information about study participants is limited. Authors are unaware of life circumstances and other confounding factors associated with anxiety and/or depression in study participants. Hence, generalizability of the study results to a larger population would require a more rigorous study to be conducted with enhanced study design and extensive data collection. Incorporation of qualitative data analysis would further strengthen the results from such study.

## Conclusion

Online meditation could be a valuable tool to combat the rising cases of anxiety and depression. This observational pilot demonstrated significant improvement with a large effect size for both anxiety and depression with 2 weeks of daily practice. Based on these promising preliminary results, the authors recommend future studies with more rigorous approaches, such as randomized control trials, to further evaluate the impact of daily Isha Kriya practice as a scalable and accessible telehealth intervention for anxiety and depression.

## Data availability statement

The raw data supporting the conclusions of this article will be made available by the authors, without undue reservation.

## Ethics statement

The studies involving human participants were reviewed and approved by Institutional Review Board of Beth Israel Deaconess Medical Center. The patients/participants provided their written informed consent to participate in this study.

## Author contributions

BS, SH, PR, RV, and SS conceptualized the study's design. PR, SH, AR, and PB conducted the data collection. PR, HO, SH, BS, RV, and PB carried out the data analysis. PR and AR did project administration and supervision. All authors contributed to the writing, review, and editing, confirmed that they had full access to all the data in the study, and accepted the responsibility to submit for publication.

## Funding

This study was entirely funded by the Sadhguru Center for a Conscious Planet, at Beth Israel Deaconess Medical Center, Boston, MA.

## Conflict of interest

The authors declare that the research was conducted in the absence of any commercial or financial relationships that could be construed as a potential conflict of interest.

The reviewer VR declared a shared affiliation with the authors SH, PR, AR, HO, PB, and BS at the time of review.

## Publisher's note

All claims expressed in this article are solely those of the authors and do not necessarily represent those of their affiliated organizations, or those of the publisher, the editors and the reviewers. Any product that may be evaluated in this article, or claim that may be made by its manufacturer, is not guaranteed or endorsed by the publisher.
