# Supplementary material for: Online guided meditation training (Isha Kriya) improves self-reported symptoms of anxiety and depression within 2 weeks of practice—An observational study
Source: Front Psychiatry. 2022 Sep 23;13:944973. doi: 10.3389/fpsyt.2022.944973 (PMC9539931; doi:10.3389/fpsyt.2022.944973)
Supplement: Supplementary file 1 [file Data_Sheet_1.docx]

**Supplement A**

**Supplement Table A1**

|  | Baseline  n = 82 | Week 2  n = 58 | Week 4  n = 37 | Week 6  n = 29 |
| --- | --- | --- | --- | --- |
| Age n (%) |  |  |  |  |
| 18-29 | 5 (6%) | 4 (7 %) | 1 (%) | 0 (%) |
| 30 - 44 | 24 (29%) | 16 (28%) | 12 (32%) | 9 (31%) |
| 45-64 | 38 (46%) | 28 (48 %) | 20 (54%) | 17 (59%) |
| 65+ | 15 (18%) | 10 (17%) | 4 (11%) | 3 (10%) |
| Gender n (%) |  |  |  |  |
| Male | 14 (17%) | 10 (17%) | 6 (16%) | 4 (14%) |
| Female | 68 (83%) | 48 (83%) | 31 (84%) | 25 (86%) |
| Other |  |  |  |  |
| Past meditation Experience n (%) |  |  |  |  |
| Yes | 54 (66%) | 36 (62%) | 25 (68%) | 21 (72%) |
| No | 28 (34%) | 22 (38%) | 12 (32%) | 8 (28%) |

**Supplemental Table A1:** Demographic information for participants for each timepoint.

**Supplement Table A2**

| **Anxiety** | **Baseline** | **Week2** | **Week 4** | **Week 6** |
| --- | --- | --- | --- | --- |
| **Mean (SD)** | 24.8 (6.96) | 16.8 (6.02) | 16.3 (6.08) | 16.3 (6.72) |
| **Median (IQR)** | 23 (9) | 17 (11) | 15 (9) | 16 (9) |
| **P-value *** | - | < 0.01 | < 0.01 | < 0.01 |
| **Cohen’s d *** | - | 1.19 | 1.22 | 1.22 |

*P-values and effect size are compared with baseline.

**Supplement Table A2:** PROMIS anxiety scores only for those who completed all time points (n=29). Significance and effect size values are based on a paired t-test.

**Supplement Table A3**

| **Depression** | **Baseline** | **Week2** | **Week 4** | **Week 6** |
| --- | --- | --- | --- | --- |
| **Mean (SD)** | 13.7 (6.34) | 8.89 (5.50) | 8.59 (5.66) | 8.14 (5.50) |
| **Median (IQR)** | 12 (12) | 8 (9) | 6 (6) | 6 (6) |
| **P-value *** | - | < 0.01 | < 0.01 | < 0.01 |
| **Cohen’s d *** | - | 0.66 | 0.72 | 0.85 |

*P-values and effect size are compared with baseline.

**Supplement Table A3:** CESD-10 scores only for those who completed all time points (n=29). Significance and effect size values are based on a paired t-test.

**Supplement Table A4**

|  | Baseline | Week 2 | Week 4 | Week 6 |
| --- | --- | --- | --- | --- |
| Completers  N  Mean (SD)  Median (Q1, Q3) | 29  24.8 (6.86)  23 (21, 30) | 29  17.3 (6.02)  17 (8, 17) | 29  16.3 (6.08)  15 (8, 15) | 29  16.3 (6.72)  16 (8, 16) |
| Dropped  N  Mean (SD)  Median (Q1, Q3) | 217^*^  24.6 (6.93)  25 (20, 29) | 29^**^  16.4 (6.53)  16 (12, 20) | 8^***^  13.8 (5.39)  11.5 (10.5, 17) | - |
| Two independent t-test | p = 0.85 | p = 0.59 | p = 0.28 | - |

* subjects who dropped after baseline

** subjects who dropped after week 2

***subjects who dropped after week 4

**Supplement Table A4:** Analysis was performed to compared anxiety values of those who dropped and those who continued with the study. We observed no statistical difference in anxiety values between these two groups at all time points.

**Supplement Table A5**

|  | Baseline | Week 2 | Week 4 | Week 6 |
| --- | --- | --- | --- | --- |
| Completers  N  Mean (SD)  Median (Q1, Q3) | 29  13.7 (6.34)  12 (8, 20) | 29  9.28 (5.50)  8 (5, 12) | 29  8.59 (5.66)  6 (4, 10) | 29  8.14 (5.50)  6 (5, 11) |
| Dropped  N  Mean (SD)  Median (Q1, Q3) | 207^*^  15.2 (7.56)  15 (10, 21) | 29^**^  8.34 (5.94)  7 (3, 12) | 7^***^  8.57 (6.35)  7 (5, 12) | - |
| Two independent t-test | p = 0.24 | p = 0.54 | p = 0.62 | - |

* subjects who dropped after baseline

** subjects who dropped after week 2

***subjects who dropped after week 4

**Supplement Table A5:** Analysis was performed to compared depression values of those who dropped and those who continued with the study. We observed no statistical difference in depression values between these two groups at all time points.
